# Rehabilitation of homonymous hemianopia: insight into blindsight

**DOI:** 10.3389/fnint.2014.00082

**Published:** 2014-10-22

**Authors:** Céline Perez, Sylvie Chokron

**Affiliations:** ^1^Neurology, Unité Fonctionnelle Vision et Cognition, Fondation Ophtalmologique RothschildParis, France; ^2^Laboratoire de Psychologie de la Perception, Université Paris-Descartes, UMR 8242 CNRSParis, France

**Keywords:** rehabilitation, homonymous hemianopia, blindsight, plasticity, brain reorganization, cortical visual impairment, post-chiasmatic damage

## Abstract

Strong evidence of considerable plasticity in primary sensory areas in the adult cortex, and of dramatic cross-modal reorganization in visual areas, after short- or long-term visual deprivation has recently been reported. In the context of patient rehabilitation, this scientifically challenging topic takes on urgent clinical relevance, especially given the lack of information about the role of such reorganization on spared or newly emerged visual performance. Amongst the most common visual field defects found upon unilateral occipital damage of the primary visual cortex is homonymous hemianopia (HH), a perfectly symmetric loss of vision in both eyes. Traditionally, geniculostriate lesions were considered to result in complete and permanent visual loss in the topographically related area of the visual field (Huber, 1992). However, numerous studies in monkeys, and later, in humans, have demonstrated that despite destruction of the striate cortex, or even following a hemispherectomy, some patients retain a certain degree of unconscious visual function, known as blindsight. Accordingly, there have recently been attempts to restore visual function in patients by stimulating unconscious preserved blindsight capacities. Herein we review different visual rehabilitation techniques designed for brain-damaged patients with visual field loss. We discuss the hypothesis that explicit (conscious) visual detection can be restored in the blind visual field by harnessing implicit (unconscious) visual capacities. The results that we summarize here underline the need for early diagnosis of cortical visual impairment (CVI), and the urgency in rehabilitating such deficits, in these patients. Based on the research precedent, we explore the link between implicit (unconscious) vision and conscious perception and discuss possible mechanisms of adaptation and plasticity in the visual cortex.

## Introduction

Although cortical visual impairments (CVI) are frequently encountered after brain damage, they are unfortunately rarely considered in neuro-rehabilitation programs. Whereas traditionally, the treatment of speech, language, and motor disorders is systematic, no visual training is usually proposed to patients with CVI.

The most frequent CVI in brain-damaged patients is homonymous hemianopia (HH), a total loss of vision in the contralesional hemifield of both eyes (Zhang et al., 2006a). However, despite the frequent occurrence of HH in these patients, it is rarely diagnosed and treated. This paucity can be explained by the fact that HH is often accompanied by more obvious neuropsychological disorders (e.g., aphasia, alexia and unilateral spatial neglect), and by a common assumption on the part of health professionals that objective recovery from visual field loss is impossible. Moreover, patients suffering from visual field defects might present with consequent anosognosia or incorrectly assume that their deficit is the consequence of an ophthalmologic lesion. Consequently, these patients are either totally unaware of their deficit, as frequently observed in cortical blindness (Chokron, 2014) or repeatedly see ophthalmologists, who are rather powerless in terms of neurovisual rehabilitation.

Surprisingly, albeit visual-field rehabilitation has been neglected, a growing number of studies on brain-damaged patients have focused on the dissociation between severely impaired explicit (conscious) vision and preserved implicit (unconscious) vision. Indeed, numerous studies in monkeys or humans with retrochiasmatic lesions have shown that some visual functions can be preserved (Humphrey and Weiskrantz, 1967). These studies have mainly promoted research in the field of perception and consciousness, but have also led to development of new visual rehabilitation programs based on the hypothesis that impaired conscious vision could be restored by training residual unconscious visual capacities.

In the review presented here, we had three objectives. Firstly, we sought to provide an overview of hemianopia and of its deleterious consequences on perception and daily life activities. Secondly, we devised new compensation and restoration techniques to treat visual field defects, emphasizing that remaining unconscious visual capacities are invaluable for restoring the visual field. Finally, we explored the possible cortical mechanisms behind restoration of visual function, by briefly overviewing neuroimaging studies on cortical plasticity in patients that had suffered from visual-field defects.

## Homonymous hemianopia

### Definition

**Figure 1:**
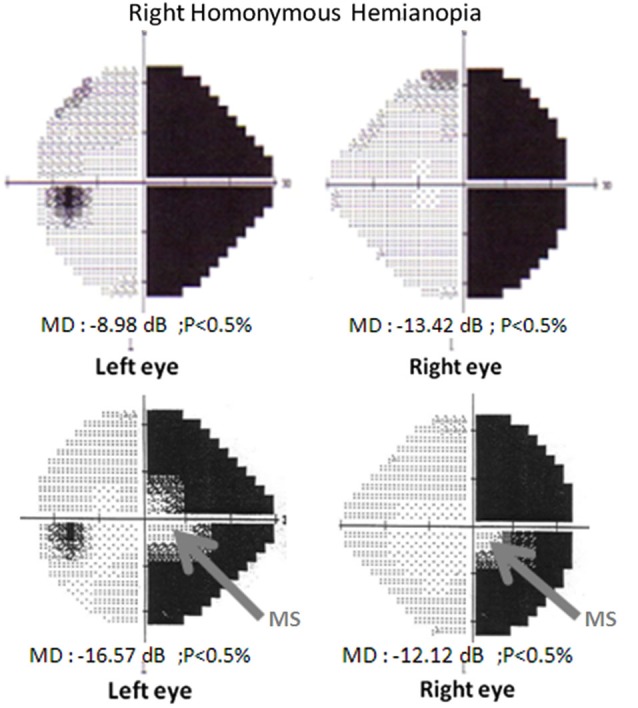
**Visual-field examination (Humphrey automated perimetry, 24-2, SITA-FAST program). Top:** Right homonymous hemianopia without macular sparing. Visual fields of the left and the right eye. **Bottom:** Right homonymous hemianopia with macular sparing. Visual fields of the left and the right eye.

The loss of vision in HH cannot be explained by injury to the eye itself (Hécaen, 1972): the lesion usually occurs in occipital regions that include to the primary visual cortex of the right or left hemisphere. HH can be total or partial (e.g., quadrantopia and scotoma) and have or lack macular sparing (if the region devoted to the central visual field is impaired), as illustrated in Figure 1 (Chokron, 1996; Danckert and Goodale, 2000). Usually, these field deficits are *totally homonymous*, meaning that the blind portion of each eye can be superimposed.

### Etiology and lesion localization

The most frequent etiology of CVI is stroke (either ischemic or hemorrhagic). According to Marshall et al. (2010), CVI, and especially visual field defects, affect more than 15% of brain-damaged patients. Moreover, in several studies researchers have proposed that 50% of the hospitalizations in neurology departments and rehabilitation centers in England are consecutive to a stroke, with 30% of these patients suffering from HH (Pambakian and Kennard, 1997; Kerkhoff, 2000; Sand et al., 2013). In one striking report, the authors indicated that among 323 stroke patients, only 8% did not show any visual impairment and 49% presented visual field loss (Rowe and VIS Group, 2009). According to a recent study, 60.5% of stroke patients might present a CVI with HH affecting 35% of stroke patients in this database of 11900 cases (Ali et al., 2013).

The aforementioned findings are testament to the urgency of visual field rehabilitation in public health. HH and other visual field defects can also result from brain tumors, cerebral hypoxia, along postchiasmatic visual pathways, occipital lobectomies, trauma, progressive multifocal leukoencephalopathy (Diller and Thompson, 2007), or even degenerative diseases (Levine et al., 1993; Chokron, 1996; Kerkoff, 1999; Zihl, 2000; Meek et al., 2013). However, it is the topography and size of the lesion, rather than the type, that determine the extent and severity of the visual field defect (Tant et al., 2002; Atchison et al., 2006). Among HH, 40% imply lesions of the occipital cortex; 30% result from parietal damage; 25%, from temporal damage; and 5%, from lesions of the optic tract or the lateral geniculate nucleus (LGN; Fujino et al., 1986; Huber, 1992).

### Clinical manifestations of HH

In addition to not being able to detect visual stimuli in their contralesional visual field, HH patients suffer from other clinical manifestations, including impaired visual search/orientation in 2D and 3D space, reading difficulties (see below), and slowed and inaccurate performance in functional visual activities (Pambakian et al., 2005; Leff et al., 2006; McDonald et al., 2006). Furthermore, numerous HH patients report locomotion disabilities, especially when outdoors: for instance, they bump into other pedestrians or obstacles in their blind hemifield. Accordingly, these patients are usually not allowed to drive. They also express difficulties in building a global representation of their visual environment (Pambakian and Kennard, 1997). Reading is considerably affected by the visual field defect: patients suffer from omission of letters (in right HH) or lines (in left HH) (Zihl, 2000). Another problem that has been described in these patients is impaired visuospatial exploration: in a study of HH and patients that were asked to generate a saccade towards the blind hemifield, the HH patients exhibited less consistent oculomotor behavior and longer fixation times (Zihl, 1995). Consequently, hemianopes suffer from disorganized, and inefficient visual search strategy that requires a lot of attention to be useful. Moreover, subjective visual assessment indicates that these patients have a reduced quality of life (Papageorgiou et al., 2007). However, and surprisingly, Papageorgiou et al. (2007) found that subjective impairment does not correlate to visual-field assessment, especially when testing the correlation between the extent of the macular spare, and the subjective evaluation of the impairment in everyday activities. This finding suggests that patients with HH should benefit from a complete evaluation including objective visual-field perimetry, evaluation of locomotion and exploration capacities as well as subjective interviews.

## Residual capacities and blindsight

Although HH patients exhibit partial or complete apparent visual loss in their contralesional visual field in objective perimetric examinations, some of them present unexpected visual capacities in their blind visual field. For example, certain patients can guide one of their hands towards a small line, according to its orientation, even though they claim that they are unaware of the stimulus (Weiskrantz et al., 1974; Perenin and Jeannerod, 1975). These capacities were referred to as *blindsight* by Weiskrantz et al. (1974). As we explain below, blindsight refers to the ability to respond to visual stimuli in the blind visual field without visual consciousness.

### Blindsight characteristics

Blindsight phenomenon has been observed in numerous tasks. Numerous experiments performed over the past several decades have shown various residual capacities in the blind field of HH patients. Using forced-choice procedures, researchers have highlighted the capacities of these patients to detect a visual stimulus placed in their blind field (Fendrich et al., 1992); to localize a visual stimulus by an eye jerk (Zihl and von Cramon, 1980) or by manual checking (Perenin and Jeannerod, 1975); to detect stimuli in movement (Riddoch, 1917) or of changing luminous intensity (Barbur et al., 1980); to discriminate among shapes (Weiskrantz, 1986); and to distinguish facial expressions (Pegna et al., 2005). For example, researchers have extensively described an HH patient named *GY* that was able to compare stimuli according to color attributes, or detect movement in his blind visual field, despite not having conscious vision of the stimuli, but that was unable to compare two degrees of luminance (Ffytche et al., 1996; Morland et al., 1999). Similar behavior has been reported in hemianopic monkeys in numerous studies by Stoerig and Cowey (1989) and Cowey and Stoerig (1995, 1997, 1999). For instance, in some studies hemianopic monkeys were able to distinguish different orientations, wavelengths and colors (for a review, see: Stoerig and Cowey, 1997; Stoerig et al., 2002). Furthermore, in hemianopic monkeys, response times towards stimuli presented in their healthy visual field can be facilitated by first displaying the stimuli in the blind field (Cowey et al., 1998).

Blindsight was not only reported for simple visual tasks such as grasping but also for tasks requiring more complex visual processing. As a matter of fact, some patients were found to be able to analyse the visual stimulus in order to perform category discrimination (Trevethan et al., 2007; Van den Stock et al., 2013, 2014) whereas de Gelder et al. (2008) were able to show that TN, a patient with cortical blindness, was able navigate and avoid obstacles although not being to report their presence. In addition, blindsight was also shown for emotional stimuli including facial and bodily expressions (e.g., Tamietto et al., 2009; Van den Stock et al., 2011) as well as for social cues such as gaze direction (Burra et al., 2013).

In a very recent study on hemianopic patients, Fayel et al. (2014) reported that these patients retained the ability to direct a saccade toward their contralesional hemifield, despite their hemifield defect, but that their verbal detection reports were at chance level. However, saccade parameters (latency and amplitude) were altered by the defect. Saccades to the contralesional hemifield in the patients exhibited longer latencies and shorter amplitudes than did those in the corresponding hemifield in a cohort of healthy subjects. Their findings confirmed previous studies on the direction of saccades in the blind field (for a review and discussion, see Cowey, 2010).

Blindsight has also been described in children. For instance, Tinelli et al. (2013) recently measured sensitivity to several visual tasks in a group of four children with congenital unilateral brain lesions that had left their optic radiations severely damaged, and in a group of three children with similar lesions that they had acquired during childhood. Using functional magnetic resonance imaging (fMRI), the authors measured blood oxygenation level-dependent (BOLD) activity in response to stimulation of each visual field quadrant. They found residual unconscious processing of position, orientation and motion of visual stimuli displayed in the scotoma in the children with the congenital lesions, but not in those with the acquired lesions.

### Neural substrates of blindsight

Visual pathways predominantly arise to the primary visual cortex (V1). Considering the global neuronal workspace (GNW) framework (Sergent and Dehaene, 2004), conscious perception would arise through the subsequent activation of several neurons, with a self-amplifying process that leads to consciousness threshold. However, if the system fails to reach said threshold, then conscious vision is not possible. Stoerig and Cowey (1995) proposed that activation of neurons in the primary visual cortex is essential for conscious vision. This premise would explain why hemianopes cannot consciously process visual information. However, visual pathways also project onto several other areas of the brain. Thus, any loss of neurons in V1 could be compensated for by the activity of other visual areas that remain stimulated by visual input. Such compensation would explain the persistence of visual capacities in the absence of conscious vision. Indeed, this idea is the central hypothesis of blindsight literature (de Gelder et al., 1999; Morland et al., 1999; Danckert and Goodale, 2000; Danckert and Rossetti, 2005; Pegna et al., 2005). Thus, researchers proposed that the underlying mechanism behind blindsight is that subcortical pathways bypass V1 to directly project onto secondary visual areas such as V5 (for motion detection), the thalamus, the brain stem, the hypothalamus, and/or the amygdala (for emotional response). In fact, this hypothesis was confirmed based on anatomical data acquired from fMRI studies. Goebel et al. (2001) identified extrastriate activations in the damaged hemisphere of the aforementioned hemianopic patient G.Y., during a forced-choice task known to elicit blindsight. Additionally, bilateral extrastriate cortex activations have been observed in several patients (Nelles et al., 2007). Unfortunately, current neuroimaging techniques are not sufficiently precise to temporally track the temporal course of visual information through subcortical pathways; consequently, the relationship between the activation of subcortical structures and blindsight is presently difficult to establish in humans (Sahraie et al., 1997).

Various hypotheses have been proposed to explain blindsight, including the presence of spared islands in V1 and the projection of visual information from the superior colliculus (SC) or LGN to preserved visual areas. We discuss these mechanisms in the following section.

#### Blindsight enabled by spared islands in V1

Given that blindsight is not observed in all hemianopic patients, some researchers have suggested that the residual visual capacities observed in some patients are enabled by spared islands in the primary visual cortex (i.e., areas that retain their function after the lesion) (Fendrich et al., 2001). However, this hypothesis has been partially disproved by reports of blindsight in patients lacking V1: in fact, patients that have undergone complete ablation of V1 can demonstrate blindsight (Perenin and Jeannerod, 1978). Moreover, in recent work based on diffusion tensor imaging (DTI), Leh et al. (2006) studied blindsight in hemispherectomy patients, ruling out the possibility of any spared islands in the primary visual cortex. They found ipsilateral and contralateral projections between the superior colliculi and primary or secondary visual areas, and frontal eye field projections, in patients exhibiting “attention blindsight”, but not in patients that did not exhibit blindsight. Moreover, in some fMRI studies, the authors did not report residual activity in the primary visual cortex in hemianopic patients (Stoerig et al., 1998). Finally, in other studies (Ptito et al., 1996), the authors observed some residual visual abilities in patients that had suffered V1 lesions, but not in patients with SC lesions. This latter finding suggests the existence of a secondary visual pathway, one that would bypass V1 to directly transmit visual information through the superior colliculi and/or the LGN. We explore the possibility of such a pathway in the next section.

#### Blindsight enabled by a secondary visual pathway that bypasses V1

The proposed secondary visual pathway would represent an alternative to the major retino-geniculo-striate pathway and entail transfer of visual information to extrastriate cortical areas through the SC or the LGN. The basis for this hypothesis is the principle observation of Riddoch (1917) that hemianopic patients can perceive and/or feel movement in their blind hemifield. He observed that patients suffering from V1 lesions could process moving stimuli but could not perceive static ones. This phenomenon, known as the* Riddoch phenomenon*, can be explained by the presence of numerous projections that extend from the superior colliculi and the pulvinar nuclei to the visual extrastriate cortex (Rodman et al., 1989). The ability of patients to plan motor actions towards visual targets that they do not consciously detect in their blind visual field corroborates the implication of such a retino-geniculo-extrastriate pathway (Weiskrantz et al., 1974; Milner, 1995). Studies on monkeys and in humans (Humphrey and Weiskrantz, 1967; Girard et al., 1992; Danckert et al., 2003), in which the dorsal pathway was found to be functioning even after destruction of V1, together indicate that dorsal structures could be involved in blindsight. Accordingly, these dorsal structures would receive afferents from subcortical areas such as the superior colliculi and the pulvinar nuclei. Indeed, Schmid et al. (2010) by testing a monkey with a damage at the level of V1 confirmed that LGN is involved in blindsight phenomenon. As a matter of fact, this study showed that the good performance in perception of high-contrasted stimuli in the blind visual field of the monkey was acompanied by a significant activation at the level of the extra-striates areas: V2, V3, V4, V5 V5/ (MT), sulcus temporal superior (FST) and the lateral parietal area (LIP), thus bypassing V1. However, following a temporary inactivation of the LGN in the lesioned hemisphere the monkey could not detect anymore the visual stimuli in its blind visual field. These results show that the direct projections from the LGN to the extra-striate cortex strongly contribute to blindsight phenomenon.

Some studies on facial categorization and judgment of facial expressions corroborate the implication of a subcortical pathway. Vuilleumier et al. (2003) reported that when subjects are shown images of faces expressing fear, their amygdala is activated via their SC. However, they observed activation of the SC and the pulvinar nuclei only when the faces expressing fear were presented at low spatial frequencies known to activate the SC. Based on this observation, the authors suggested that this subcortical pathway might provide inputs to the amygdala. In related work, Pegna et al. (2005), upon studying a patient that suffered from cortical blindness following a bi-occipital lesion, observed that the patient was able to recognize feelings on faces. This phenomenon is known as *affective blindsight* and is associated to activation of the right amygdala. Tamietto and de Gelder (2008), proposed that affective blindsight could be enabled when visual information is transmitted by the subcortical pathway to the colliculi, and further on to the amygdalae, all the while bypassing V1. Moreover, Morris et al. (2001), using fMRI, confirmed that affective blindsight can be elicited through the same subcortical pathway involving the SC, the thalamus and the amygdalae (spared by V1 damage) (for a review and discussion, see: Tamietto and de Gelder, 2010). Finally, Tamietto et al. (2010) also have provided evidence that the collicular-extrastriate pathway has a crucial role in non-conscious visuomotor integration: they showed that, in the absence of V1, the SC is essential for translating visual signals that cannot be consciously perceived, into motor outputs. They presented a gray stimulus in the blind field of patients with a unilateral lesion of V1 and observed that, although the patients did not consciously see the stimulus, it nevertheless influences their behavioral and pupillary responses to stimuli consciously seen in their intact field. The authors called this phenomena *implicit bilateral summation* because the unseen stimulus could affect the response to the seen stimulus. However, it should be noted that this effect was accompanied by activation in the SC and in occipito-temporal extrastriate areas. Interestingly, when the authors instead presented their subjects with purple stimuli (which predominantly entail S-cones and consequently, are not detected by the SC), they did not observe any evidence of implicit visuomotor integration and found a massive decline in activations in the SC. Based on these findings, the authors suggest that the SC bridges cerebral sensory and motor processing, thereby contributing to visually-guided behavior that is functionally and anatomically separate from the geniculo-striate pathway and entirely external to conscious vision. Such a scenario would partly explain blindsight.

Despite the aforementioned results, in other neuroimaging studies, the authors reported an absence of relationship between blindsight, and activation of subcortical structures. The lack of evidence highlights the limits of conventional fMRI to study blindsight. This might explain why Leh et al. (2006) used DTI, rather than conventional fMRI, in order to study the neural substrate of blindsight in hemianopic patients. They tested hemispherectomy patients with a visual field defect, seeking to exclude the presence of spared islands in the visual cortex. They observed ipsilateral and contralateral projections from the SC to the primary and extrastriate visual areas in patients with type I blindsight (attention blindsight), but not in patients that did not exhibit attention blindsight or in control subjects.

More recently, Bridge et al. (2008) described new evidence for three anatomical connections that could underlie blindsight. Firstly, control subjects and the patient GY showed a tract that bypassed V1 and connected the LGN to the ipsilateral visual motion area MT+/V5 as reported by Sincich et al. (2004) in the macaque monkey. Secondly, ipsilateral pathways between MT+/V5 and LGN were found in GY lesioned and intact hemispheres as controls. Finally, they found two other pathways in GY but not in the controls: the first one crossed white-matter tracts that connect the LGN to the contralateral MT+/V5 (i.e., contralateral tracts between the LGN in one hemisphere and MT+/V5 in the other), through the splenium; and the second one was a transcallosal connection between the MT+/V5 areas in each hemisphere. These specific connections found in GY are consistent with a contralateral pathway from right LGN to left MT+/V5, and with the increased inter-hemispheric transfer of information found in several studies (Goebel et al., 2001; Silvanto et al., 2007; Bridge et al., 2008). However, whether the emergence of new connections results from strengthening of existing pathways, or from development of new pathways, remains to be determined. Moreover, since GY was 8 years old at the moment of his lesion, he had a greater likelihood of regeneration of connections than would an adult patient. Very recently, also using DTI, Tamietto et al. (2012) suggested that selective changes may occur in patients with CVI in the SC, the pulvinar and the amygdala. The authors suggested that these changes may explain the residual sensitivity to emotions or social signals in blindsight (Tamietto et al., 2012).

In summary, blindsight might be enabled by retinotectal projections that bypass V1 and could result from connections specific to it. However, its dynamics remain scarcely understood. Recently, Ioannides et al. (2012) used magnetoencephalography to study the spatiotemporal profiles of visual processing and the causal contribution of V1 in three neurologically intact participants and in GY in whom residual visual functions mediated by the extra-geniculostriate pathways have been reported. Whereas normally perceived stimuli in the left hemifield of GY elicited a spatiotemporal profile in the intact right hemisphere that closely matched that of healthy subjects, stimuli presented in his contralesional hemifield produced no detectable response during the first phase of processing. The authors reported that in contrast to responses in the intact hemisphere, the back-propagated activity in the early visual cortex did not exhibit the classic retinotopic organization and did not have well-defined response peaks thus suggesting a modification in the spatiotemporal profiles of visual processing after a unilateral destruction of V1.

Below we present the spontaneous recovery of HH, and then examine the various rehabilitation techniques that have been proposed to compensate for and/or reduce this related visual-field defect.

## Spontaneous recovery of HH

Patients can spontaneously recover from HH, but the probability of such recovery is proportional to the time that has elapsed since the lesion occurred. Reported recovery rates range from 7% to 86% (for a review, see: Sabel and Kasten, 2000). Furthermore, a large area of residual vision is a better predictor of spontaneous recovery following stroke than is a small area. In a 15-year longitudinal study, Zhang et al. (2006b) analyzed spontaneous recovery in hemianopia patients. They observed recovery approximately 38.4% of the cases within the commonly accepted period of 6 months (after which, the HH becomes chronic). The chance of recovery diminished with increasing time since injury: at 1 month, the rate was > 50%, whereas at 6 months, it was only 20%. Moreover, in most cases, the recovery was very limited: the patients had only regained a few degrees of vision; indeed, only 5.3% of the patients had completely recovered from their visual field defect. Some HH patients adopt spontaneous compensations, especially in terms of exploration strategies, that are quite different from those in non-hemianopic subjects (Pambakian et al., 2004). Sabel and Kasten (2000) considered that after 3–6 months post-lesion, partially recovered patients can only achieve further improvement through visual capacities training.

## Rehabilitation of HH patients

Although HH does not seem to be as debilitating as spatial neglect, it can seriously affect daily activities such as driving, walking in crowded areas, crossing the street, and reading. Problems with these and other activities can pose serious problems for recovering HH patients upon their return to work. Given the poor rate of spontaneous recovery in HH, several training programs have been proposed to help patients recover. These programs can be classified into three categories according to their objectives for the visual-field deficit: *substitution, compensation* and* recovery*.

### Substitution therapies

An early but now-defunct substitution technique entailed the use of optical aids (e.g., mirrors or Fresnel prisms) to shift visual information from the blind visual field to the central or the ipsilesional, preserved visual field. However, there were only anecdotal reports of the positive effects of this approach; furthermore, such tools were reported to cause diminished acuity, confusion or even diplopia in patients (for a review, see: Pambakian and Kennard, 1997; Grunda et al., 2013). Therefore, this technique is no longer used in rehabilitation of HH patients.

Currently, compensatory techniques for HH are used principally to enlarge and reinforce visual search, by training patients in oculomotor strategies. Indeed, there are extensive reports that hemianopic patients have difficulties in visual scanning for object detection, as well as in identifying people. These problems can lead to omission of important parts of a scene and consequently, to poor comprehension and to social misunderstanding. Parafoveal visual-field defects also affect reading (*hemianopic alexia*), due to a reduction in the “perceptual window” involved in letter identification and in saccades planification and thus compromizing guidance of eye movements along text (Poppelreuter, 1971[1990]; Zihl, 1995; McDonald et al., 2006). These deficits can selectively be trained in hemianopic patients (Schuett et al., 2012).

### Compensation therapies

Compensation therapies can be proposed regarding the fact that recovery from a very severe perceptual deficit can be difficult to obtain. Therefore, they typically involve using and modifying the patient’s preserved capacities to sidestep the impairment or render it less disabling. Accordingly, compensation strategies for HH require the use of the ipsilesional hemifield or of the central visual field to compensate for the blind area in the contralesional hemifield.

In classical oculomotor rehabilitation, patients are trained to search for a stimulus projected into their blind hemifield, and then respond to it as quickly as possible. The target can be presented alone or amongst distracters (Zihl and Werth, 1984). Researchers usually record reaction times (RTs) and error rate, given that an inefficient search will lead to longer RTs. These techniques are generally efficient, with shorter RTs, but sometimes lead to longer RTs (Pambakian et al., 2004). Although longer RTs could be considered as being detrimental, in some cases, they have been described as an improvement: the authors of these studies affirm that longer RTs might reflect underlying compensatory mechanisms (i.e., the development of a new strategy) that probably need more time to progress and reach maximum efficacy. These types of compensation techniques are principally based on *top-down* mechanisms, because they train patients to focus their attention on the blind hemifield.

Another compensation technique involves a *bottom-up* strategy based on multisensory stimulation and integration (Bolognini et al., 2005). According to these authors, this therapy does not require the patient’s attention, which can be impaired in some patients and therefore, enables more interesting perspectives for rehabilitation. They based their rehabilitation technique on the existence of neurons that encode information coming from different sensory modalities, in the superior colliculi and other parts of the brain. As the superior colliculi are involved in gaze orientation, and the visual modality is impaired in these patients, Bolognini et al. proposed that a non-visual modality (e.g., auditory) could be harnessed to reinforce gaze orientation towards the blind hemifield. They presented their patients with audio-visual stimulation to help them find a subsequently presented visual target. The authors observed a greater increase in visual oculomotor exploration upon the addition of an auditory cue together with the visual stimulus than without it. Thus, multisensory stimulation enabled the capacities of a fully functioning sense (hearing) to be transferred to a deficient sense (vision).

Finally, other researchers have focused on the reading impairment caused by HH. For instance, Spitzyna et al. (2007) induced an optokinetic nystagmus in order to facilitate reading in hemianopic patients. They presented patients with right-to-left moving text that the patients had to read. The right-to-left visual movement induced a left-to-right nystagmus that increased reading speed.

Although the aim of these compensatory techniques is not to restore *per se* the impaired visual field, they nevertheless improve the quality of life of patients. Indeed, overall, in these studies the authors report subjective improvement in everyday life, according to subjective questionnaires (Bolognini et al., 2005). Therefore, compensation therapies seem to be a first step that should be systematically proposed for hemianopia treatment when restoration therapy is unavailable. However, and as we previously mentioned, since these techniques are compensatory in nature, the visual impairment remains. In addition, there are reports that the ipsilesional hemifield of hemianopic patients, when thought to be intact, can actually also be impaired—not in terms of visual-field loss, but in terms of quality of vision (Paramei and Sabel, 2008; Bola et al., 2013). Thus, relying exclusively on the “intact” visual field may not be the best option in rehabilitation techniques. Furthermore, special care for visual-field improvement, and restoration therapies of the blind hemifield, are needed whenever possible. In the following section we discuss restoration therapies.

### Restoration therapies

Attempts to enlarge the visual field appear to be a more encouraging way to help hemianopic patients. Unfortunately, as we explained in the previous section, restoration of visual function may seem impossible in HH patients. This might be linked to the fact that, even if a few studies demonstrated in particular condition (see above) that visual experience is possible even in the absence of V1, generally V1 is considered to be crucial for visual consciousness (Weiskrantz et al., 1974; Perenin and Jeannerod, 1975; Tamietto et al., 2010).

Thus, recovery of explicit conscious visual detection in the absence of V1 would imply a degree of neural plasticity that is known to be unattainable after a normal rearrangement during the first 3 months post-lesion. Moreover, whether any reorganization that did occur would be powerful enough to activate conscious vision is unknown. However, studies in animals and humans have shown that perceptual learning is possible in hemianopia (i.e., training can improve visual perception) (Fahle and Poggio, 2002). A few researchers have hypothesized that the size of the visual field could be increased to enable recovery from blindness (Chokron et al., 2008). In the next section we describe oculomotor rehabilitation techniques, followed by restorative techniques using blindsight.

#### Oculomotor rehabilitation techniques

Most of these studies derive from the use of compensatory techniques: the authors found that training HH patients to direct saccades towards the border zone of their blind field could partially increase the size of their visual field (van der Wildt and Bergsma, 1997). These results led to the development of a special training platform, called Visual Restoration Therapy (VRT; Sabel and Kasten, 2000), to enlarge the size of the isolated patches of residual vision in the blind hemifield. Patients were trained at home, on their TV screen, with a computerized program that is adapted to their visual capacities and evolves according to their progresses. They were asked to fix their gaze in the center of the screen, and trained to detect a target placed in the border zone of their hemianopic field, by pressing a button whenever they saw it (Sabel et al., 2005; Kasten et al., 2006). Partial improvement was found in some of the studies. For example, in one study, 15 patients trained with VRT for 3 months showed only a 3.8% increment in stimulus detection (Kasten et al., 2006). In a recent, larger and longer-term study, in which 302 patients were trained with VRT for 6 months (Mueller et al., 2007), 17.2% of the patients exhibited increased detection of supra threshold stimuli, although 29.1% did not show any improvement. However, the field extension averaged 4.9 ± 0.41°. Subjective assessment showed improved visual confidence in 75.4% of the patients. Overall these authors have been criticized for a fastidious program that would induce only weak improvement (Glisson and Galetta, 2007). Horton (2005) explained that patients showed subjective improvement, and emphasized that this improvement was only measured with the device that was used for therapy. Indeed, when tested on classical automatic perimeter, no objective enlargement of the visual field was recorded.

A training device named the Lubeck Reaction Perimeter was created by Schmielau (1996), and then evaluated by Schmielau and Wong (2007) in a study of 20 patients that were trained twice a week for an average of 8.2 months. The patients were seated in front of a large size hemispheric half-bowl filled with LEDs that would light up according to their visual capacities, as in the VRT. The patients were asked to fixate on the red central LED during testing. The results were interesting: 17 out of the 20 patients exhibited an improvement in detection rate in their impaired field (average rate: 11.3° ± 8.1). However, among the drawbacks of the Lubeck Reaction Perimeter are its large size and the fact that it is not amenable to home use (Sabel, 2008).

A completely different approach to that used in the aforementioned devices is one based on that hypothesis that conscious visual detection could be restored by training unconscious visual processing capacities (i.e., blindsight) in hemianopia.

#### From blindsight to sight

As we mentioned above, some patients can actually perform visual tasks in their impaired hemifield, despite claiming that they cannot see or feel anything (Weiskrantz et al., 1974). Although Ducarne and Barbeau (1981) and Ducarne de Ribaucourt and Barbeau (1993) did not relate their visual training to blindsight, they were the first authors to report the need to stimulate the blind visual field by using tasks of localization and detection of salient visual targets associated with prehension. Indeed, Danckert et al. (2003) subsequently proposed that the driving action directed in the defective visual field enables strengthening of visual perception. Thus, asking patients to perceive, judge, recognize, locate or grasp stimuli in their hemianopic visual field could help them to “relearn” how to see.

Although blindsight had been extensively studied from the theoretical and experimental perspectives over the past few decades, only in the past 15 years did a few authors begin to hypothesize that blindsight capacities could be improved through training (Sahraie et al., 2006) and therefore, that unconscious vision might be transformable into conscious vision (Ro and Rafal, 2006). Firstly, Zihl (2000) observed that patients that had been trained to locate targets in their blind hemifield gained subjective improvements in sight for everyday life activities. More recently, Sahraie et al. (2006) claimed that visual sensitivity in the blind hemifield could be improved without the patient’s awareness, and that this could be done even in the very depth of the impaired hemifield (and not only in the border zone of blindness, as suggested in previous studies). The authors “trained” 12 patients to discriminate grating stimuli from non-grating ones in their blind hemifield over 3 months. Before and after the treatment, they tested the patients for detection of various levels of contrasts and spatial frequencies, clinical perimetry, and a subjective estimate of the visual-field defect. Overall, the patients exhibited improved sensitivity in target detection at various contrast sensitivity levels and spatial frequencies, and objective improvements at clinical perimetry tests. Therefore, authors concluded that training blindsight could be a way to improve visual field defect in hemianopia. In related work, Raninen et al. (2007) trained patients to detect flickering stimuli and to discriminate letters at various eccentricities within the blind hemifield. The patients were tested twice weekly for roughly 1 year, ultimately improving in these tasks, but not in Goldman perimetry.

Considering that objective improvement has been reported in some studies on blindsight training, and that the use of tasks that are more ecological might help patients to recover, Chokron et al. (2008) trained nine brain damaged patients on various forced-choice tasks in their blind hemifield, including visual-target pointing, letter recognition, comparison of two stimuli presented in both hemifields and target location. Such tasks are usually proposed to test for blindsight. Patients took pre- and post-tests (letter identification and target detection), and were evaluated with an automated perimetry exam. They all improved in behavioral tasks, and eight of the nine patients exhibited a significant enlargement of their visual field (as determined by classical perimetry examination). These findings suggest that stimulating blindsight can help the patients to be aware of unconscious perceptions that typically remain unknown and unused by the patient if not trained. Furthermore, Sahraie et al. (2013) recently tested five HH patients on a forced-choice detection task, observing improvement in four of the patients. With repeated stimulation, detection in the hemianopic field improved.

Interestingly, therapies visual or blindsight stimulation in the contralesional visual field are presently being evaluated in children with visual-field defects, consecutive to perinatal asphyxia, or traumatic brain injury (Dutton and Bax, 2010; Pawletko et al., 2014). However, no clinical trials have yet been performed in this area.

The possibility of visual-field restoration raises the question of whether some type of cortical reorganization occurs after a unilateral occipital lesion. As we explain below, further neuroimaging studies on patients before and after blindsight stimulation are required in order to understand how vision might be regained.

## Cortical reorganization after an occipital lesion

### Cortical reorganization in hemianopic patients

Plasticity is a fundamental mechanism for the brain to adjust to sensory changes in the surroundings, to improve perception and to recover from damage to the visual system. Studies on neural plasticity have shown that the brain can react to environmental inputs after a lesion, in infants, children and even seniors. Thus, researchers have demonstrated that part of the areas adjacent to a lesion can replace the function of the affected one, as can occur after sensory-motor defects (Liepert et al., 2000), or that the healthy (undamaged) hemisphere can reorganized itself to take over part of the damaged hemisphere’s functions (Netz et al., 1997; Johansen-Berg et al., 2002; Rossini and Dal Forno, 2004). In related work, Safran and Landis (1999) suggested that even in adult brains, cortical maps are not fixed.

Studying blindsight enables researchers to determine, in the absence of the primary visual system, whether the spared or recovered visual ability stems from an existing alternative pathway or from newly formed pathway. As described above, Bridge et al. (2008), using DTI, and Silvanto et al. (2009), using transcranial magnetic stimulation (TMS), each observed pathways in patients with visual-field defects that they did not observed in control patients. Their findings suggest that after a brain lesion, specific connections can be created. Nelles et al. (2007) conducted a related study in thirteen hemianopic patients that had suffered a unilateral ischemic stroke in the striate cortex. Stimulation in the blind visual field led to bilateral activation in the extrastriate areas, and this activation was stronger in the ipsilateral (healthy) hemisphere. This result suggests that activation had been transferred from the damaged hemisphere to the healthy one.

Interestingly, in most of the published studies concerned with cortical reorganization in hemianopic patients, the authors did not address the side of the lesion. This issue was tackled by Perez et al. (2013), who sought to determine the effects of the lesion side on cortical reorganization in brain-damaged patients. They demonstrated that the pattern of cortical activation during a visual detection task and a visual categorization task depends on the side of the occipital lesion. Indeed, RHH patients showed activation predominantly in the right (intact) hemisphere (occipital lobe and posterior temporal areas), whereas LHH patients showed more bilateral activation (in the occipital lobes). Thus, lateralization of an occipital lesion is not without consequence for the subsequent cortical reorganization. Along those lines, the impact of the lesion lateralization might be crucial to the cortical reorganization and consequently, to the choice of rehabilitation scheme for left or right hemianopic patients.

The main question when studying brain reorganization after recovery is: *Does recovery involve recruitment of existing pathways, establishment of new neural connections or both?* We address this question in the following section.

### Cortical reorganization in hemianopic patients after visual recovery

As explained above, various techniques are now available to compensate for damage, or restore vision, in the hemianopic visual field. Regarding the functional basis of these rehabilitation techniques, some studies have suggested that visual training can induce neural modifications in V1 (Furmanski et al., 2004; Maertens and Pollmann, 2005). For example, Raninen et al. (2007) demonstrated that intensive visual training can improve abilities in the blind visual field. After training, their patients achieved a level of performance in the blind visual field similar to that in the healthy visual field. Furthermore, this gain was underlined by changes in cortical activation. Indeed, after training, the patients exhibited an ipsilateral response to blind visual-field stimulation. In a related fMRI study, Marshall et al. (2008) were the first to report changes in cerebral responses to stimuli after visual training in hemianopic patients. They trained the patients with repetitive stimulation of the border zone. After 1 month of training, the patients exhibited increased BOLD activity for border-zone detection compared to detection in the healthy part of the visual field. After visual training, the most activated areas were the right inferior and lateral temporal areas, the right dorsolateral frontal areas, the bilateral anterior cingulate cortices and the bilateral basal ganglia. Moreover, these authors observed a correlation with behavioral data obtained out-of-scanner, which revealed an improvement in response times for detecting stimuli in the border zone. The authors concluded that visual training induced a shift of attention from the non-trained seeing field to the trained border zone, and stated that the effect seemed to be mediated by frontal regions and other higher-order visual areas. In related work, Ro and Rafal (2006) trained patients, and then observed an ipsilateral response to blind visual-field stimulation. They proposed that the visual pathway used in blindsight—in this case, the sub-cortical pathway—is probably the same one that underpins recovery after training.

Henriksson et al. (2007) trained patients, and then studied them by fMRI, observing that visual information in both hemifields was processed in the intact hemisphere. Thus, training patients for visual detection in the hemianopic field induced cortical representation of this visual field in their ipsilateral, healthy hemisphere. As such, after training, the visual areas of the healthy hemisphere (in particular, V5) might have represented not only the contralateral field, but also the ipsilateral visual field. This premise is consistent with the findings of Nelles et al. (2007), in their study of hemianopic-field stimulation in patients with occipital strokes: they found that stimulation induced bilateral activation within the extrastriate cortex, and this activation was stronger in the ipsilateral (contralesional) hemisphere. Furthermore, the activation pattern in the patients was different to that observed in normal subjects, who exhibited contralateral activation of the striate and extrastriate regions (as similarly observed by Silvanto et al., 2007, using TMS).

Recently, Plow et al. (2012) studied a 3-month regime of VRT in two patients suffering from a left occipital lesion (Right HH). They coupled sessions of visual training with TMS of the occipital area. They observed better recovery in the patients (who had received TMS) compared to control patients (who had received sham stimulations). Using fMRI, they also recorded activation around the lesioned area and bilateral activation of the associative visual areas.

Taken together, the aforementioned studies suggest that visual training in the blind visual field, regardless of whether it is coupled to TMS, can induce cortical reorganization. Nevertheless, more studies are needed in order to standardize these rehabilitation programs as well as to understand the neural bases of recovery. Furthermore, psychophysical studies are required to determine the extent to which the recovered vision in the hemianopic visual field resembles the vision in the ipsilesional field.

## Conclusion

Since hemianopia involves impairment in early perceptual processes, recovery from it was once considered impossible. However, recent findings on blindsight offer new perspectives for recovery of visual function in patients with a post-chiasmatic damage. From a theoretical point of view, we still need to understand how training unconscious visual capacities can lead to a restoration of conscious visual capacities. According to the GNW model (Dehaene and Changeux, 2011) conscious access occurs when incoming information is made globally available to multiple brain systems through a network of neurons with long-range axons densely distributed in prefrontal, parieto-temporal, and cingulate cortices. We still need to understand how stimulating blindsight can promote conscious perception. However, one can hypothesize that responding to a stimulus, although without being conscious of it, may in turns, activate parts of this network and in this way induce conscious access. As a matter of fact, Sergent et al. (2013) recently tested the influence of postcued attention on perception, using a single visual stimulus (Gabor patch) at threshold contrast in healthy participants. The authors showed that postcued attention can retrospectively trigger the conscious perception of a stimulus that would otherwise have escaped consciousness. One can thus hypothesize that acting on a stimulus which was not consciously detected might act as a postcue and thus produce conscious perception in the blind field of hemianopic patients. Along those lines, stimulating blindsight could help restoring conscious perception.

Nevertheless, further testing is required in order to confirm the findings that we have summarized here as well as to explain how they help vision recovery. Furthermore, rehabilitation programs should be generalized and standardized in order to facilitate identification of those patients that are the best candidates for treatment and to enable better treatment. Therefore, and as has been proposed for rehabilitation of spatial neglect, we propose that rehabilitation of hemianopia should include a combination of compensatory and restorative techniques tailored to each patient. Indeed, the majority of studies have shown that the use of both types of techniques should help patients to make improvements in everyday life activities.

Future research on blindsight should include neuroimaging studies. One interesting line of research would be to study the link between neural activation and post-training recovery in HH patients. Another interesting study would be to use fMRI to compare HH patients during the acute phase and after training, once they have recovered their field of vision, in order to understand the neural substrates of visual recovery. Finally, researchers should also compare HH patients that have recovered vision to those that have not, in order to determine if the two groups exhibit the same cortical reorganization.

## Conflict of interest statement

The Guest Associate Editor Olivier A. Coubard declares that, despite having collaborated in the past with authors Céline Perez and Sylvie Chokron, the review process was handled objectively and no conflict of interest exists. The authors declare that the research was conducted in the absence of any commercial or financial relationships that could be construed as a potential conflict of interest.
